# Supplementary material for: Benefits of Adhering to a Mediterranean Diet Supplemented with Extra Virgin Olive Oil and Pistachios in Pregnancy on the Health of Offspring at 2 Years of Age. Results of the San Carlos Gestational Diabetes Mellitus Prevention Study
Source: J Clin Med. 2020 May 13;9(5):1454. doi: 10.3390/jcm9051454 (PMC7290358; doi:10.3390/jcm9051454)
Supplement: Supplementary file 1 [file jcm-09-01454-s001.pdf]

Table 1. Children's data at 2 years follow-up according to whether their mothers belonged to the intervention or control group, and subdivided according to glucose tolerance (GDM or NGT).

	**Control Group (*n*=365)**			**Intervention Group (*n*= 338)**			IG vs. CG	
	**NGT**	**GDM**	**p[a]**	**NGT**	**GDM**	**p[a]**	**p[b]**	**p[c]**
n	274	91		280	58			
Age (months)	23.2 ± 2.5	22.9 ± 2.8	0.287	23.3 ± 2.4	23.5 ± 2.9	0.682	0.871	0.261
Body Weight (Kg)	12.1 ± 1.5	12.2 ± 1.6	0.575	12.2 ± 1.5	11.9 ± 1.7	0.224	0.255	0.349
percentile	46.3 ± 27.3	48.3 ± 30.2	0.428	49.6 ± 27.6	45.9 ± 26.3	0.325	0.155	0.642
Height (cm)	86.4 ± 3.9	85.9 ± 4.0	0.306	86.3 ± 4.0	85.6 ± 4.1	0.282	0.746	0.683
Percentile	38.9 ± 27.8	40.7 ± 29.5	0.144	40.4 ± 29.0	36.9 ± 26.5	0.236	0.544	0.477
Breastfeeding *n* (%)	255 (94.1)	83 (94.3)	0.588	261 (93.9)	53 (91.4)	0.324	0.530	0.356
Exclusive(months)	5.2 ± 1.5	5.2 ± 1.4	0.989	5.4 ± 1.4	4.9 ± 1.8	0.073	0.078	0.406
Mixed (months)	10.5 ± 7.7	9.9 ± 7.8	0.627	10.5 ± 7.2	11.2 ± 9.3	0.667	0.979	0.489
Cereal Introduction (months)								
Gluten-free cereal	4.8 ± 0.8	4.8 ± 0.8	0.632	4.8 ± 0.8	4.6 ± 0.8	0.436	0.935	0.331
Gluten cereal	6.6 ± 1.3	6.6 ± 1.3	0.832	6.5 ± 1.7	6.9 ± 3.2	0.399	0.864	0.483
Nursery								
n (%)	185 (67.5)	62 (68.1)	0.511	194 (70.0)	43 (74.1)	0.325	0.293	0.276
Age (months)	15.6 ± 6.3	16.2 ± 5.3	0.530	14.8 ± 6.8	14.7 ± 6.9	0.933	0.281	0.302
Vaccinations *n* (%)								
Compulsory	268 (99.3)	91 (100)	0.559	280 (100)	58 (100)	1	0.245	1
Recommended *n* (%)								
Meningitis	155 (57.4)	55 (60.4)	0.352	179 (65.1)	35 (60.3)	0.294	0.040	0.583
Rotavirus	183 (67.8)	68 (74.7)	0.132	203 (73.8)	37 (63.8)	0.085	0.073	0.108
Outpatients diseases *n* (%)								
Treatment with antibiotics	189 (69)	62 (68.1)	0.489	192 (68.8)	42 (72.4)	0.355	0.520	0.357
Treatment with corticosteroids	141 (51.5)	46 (50.5)	0.488	137 (49.1)	29 (50.0)	0.508	0.320	0.541
Diagnoses *n* (%)								
Food allergies	21 (7.7)	8 (8.8)	0.447	17 (6.1)	4 (6.9)	0.503	0.279	0.466
Asthma	7 (2.6)	0 (0)	0.131	7 (2.5)	4 (6.9)	0.102	0.590	0.022
Bronchiolitis/respiratory problems	57 (20.9)	17 (18.7)	0.387	64 (22.9)	11 (19.0)	0.319	0.315	0.564
Atopic dermatitis	77 (28.2)	28 (30.7)	0.424	82 (29.4)	20 (34.4)	0.053	0.416	0.092
Severe Diseases inpatients treatment *n* (%)								
All-cause hospital stays	51 (18.6)	14 (15.4)	0.299	39 (13.9)	12 (20.7)	0.135	0.084	0.289
Children	48 (17.5)	14 (15.4)	0.399	36 (12.9)	12 (20.7)	0.236	0.464	0.504
Duration (days)	13.4 ± 28.3	6.7 ± 6.2	0.287	4.2 ± 2.7	15.3 ± 16.3	0.003	0.121	0.231

Bronchiolitis/asthma disease	24 (8.8)	3 (3.3)	0.050	14 (5.0)	4 (6.9)	0.376	0.046	0.265
Treatment with antibiotics	46 (16.8)	13 (14.3)	0.352	31 (11.1)	9 (15.5)	0.230	0.035	0.507
Treatment with corticosteroids	34 (12.4)	7 (7.7)	0.134	19 (6.8)	6 (10.3)	0.246	0.018	0.391
Treatment with antibiotics and corticosteroids	30 (10.9)	6 (6.6)	0.157	19 (6.8)	6 (10.3)	0.246	0.059	0.300

Results expressed as mean ± SD or *n* (%).GDM, gestational diabetes mellitus; NGT, normal glucose tolerance. P differences between groups analyzed with the χ^2 test (categorical variable); Student's t test (continuous variables) or the Mann–Whitney U test (not-normal distribution in continuous variables). Verified by the Shapiro-Wilk test. p^a: compares NGT to GDM in each group (CG and IG); p^b: compares women with NGT between groups; p^c, compares women with GDM between groups.

Table 2. Children's data at 2 years follow-up according to whether their mothers belonged to the intervention or control group, and subdivided according to BMI (<25kg/m² or ≥25kg/m²).

	**Control Group (*n*=365)**			**Intervention Group (*n*= 338)**			**IG vs. CG**	
	**BMI <25 Kg/m²**	**BMI ≥25 Kg/m²**	**p^a**	**BMI <25 Kg/m⁻²**	**BMI ≥ 25 Kg/m²**	**p^a**	**p^b**	**p^c**
n	258	107		252	86			
Age (months)	23.2 ± 2.5	23.1 ± 2.7	0.681	23.3 ± 2.6	23.2 ± 2.4	0.734	0.582	0.711
Body Weight (Kg)	12.0 ± 1.4	12.4 ± 1.6	0.068	12.1 ± 1.5	12.4 ± 1.7	0.223	0.459	0.909
percentile	46.2 ± 27.7	51.4 ± 28.8	0.105	47.6 ± 27.5	54.5 ± 26.4	0.057	0.561	0.458
Height (cm)	86.3 ± 3.9	86.1 ± 4.0	0.667	86.2 ± 4.1	86.2 ± 3.8	0.981	0.633	0.948
percentile	38.2 ± 28.0	41.5 ± 27.9	0.315	38.5 ± 28.7	43.7 ± 27.8	0.188	0.894	0.606
Breastfeeding *n* (%)	244 (95.7)	89 (89.9)	0.039	237 (94.0)	77 (91.7)	0.296	0.263	0.441
Exclusive (months)	5.2 ± 1.5	5.3 ± 1.4	0.688	5.3 ± 1.5	5.5 ± 1.4	0.308	0.408	0.280
Mixed (months)	10.1 ± 7.6	10.9 ± 8.1	0.477	10.7 ± 7.6	10.3 ± 7.3	0.709	0.486	0.629
Cereal Introduction (months)								
Gluten-free cereals	4.8 ± 0.8	4.7 ± 0.8	0.281	4.7 ± 0.8	4.8 ± 0.8	0.556	0.102	0.515
Gluten	6.5 ± 1.3	6.7 ± 1.2	0.243	6.6 ± 1.9	6.7 ± 2.5	0.902	0.634	0.823
Nursery								
n (%)	179 (69.4)	64 (62.7)	0.139	173 (68.7)	64 (77.1)	0.090	0.467	0.025
Age (months)	15.5 ± 5.7	16.4 ± 7.0	0.406	14.9 ± 6.8	14.6 ± 7.0	0.801	0.405	0.186
Vaccinations *n* (%)								
Compulsory	258 (100)	100 (98.0)	0.082	252 (100)	84 (100)	1	1	0.308
Recommended *n* (%)								
Meningitis	161 (63.4)	48 (47.1)	0.003	172 (68.5)	42 (50.0)	0.004	0.040	0.340
Rotavirus *n* (%)	186 (73.2)	61 (59.8)	0.010	188 (74.9)	52 (63.4)	0.032	0.372	0.365
Outpatients diseases *n* (%)								
Treatment with antibiotics	177 (68.6)	69 (67.6)	0.477	182 (71.9)	52 (51.9)	0.057	0.234	0.259
Treatment with corticosteroids	128 (49.6)	56 (54.9)	0.216	123 (48.6)	43 (51.2)	0.389	0.446	0.360

Food allergies	23 (9.0)	6 (5.9)	0.228	17 (6.7)	4 (4.8)	0.364	0.216	0.500
Asthma	2 (0.8)	5 (4.9)	0.022	7 (2.8)	4 (4.8)	0.283	0.084	0.620
Bronchiolitis/respiratory problems	50 (19.5)	22 (21.6)	0.376	54 (21.3)	21 (25.0)	0.289	0.337	0.352
Atopic dermatitis	71 (27.6)	28 (27.5)	0.542	82 (32.4)	24 (28.6)	0.303	0.140	0.497
Severe Diseases inpatients treatment *n* (%)								
All-cause hospital stays *n* (%)	50 (19.4)	14 (13.7)	0.132	33 (13.0)	18 (20.9)	0.048	0.033	0.117
Children *n* (%)	49 (19.0)	13 (12.1)	0.256	30 (11.9)	18 (20.9)	0.461	0.508	0.537
Duration (days)	14.1 ± 28.6	5.6 ± 6.9	0.172	6.1 ± 6.9	7.7 ± 11.6	0.670	0.199	0.552
Bronchiolitis/asthma diseases	21 (8.2)	6 (5.9)	0.306	11 (4.3)	7 (8.4)	0.124	0.043	0.348
Treatment with antibiotics	44 (17.1)	14 (13.7)	0.268	28 (11.0)	12 (14.5)	0.255	0.033	0.526
Treatment with corticosteroids	34 (13.2)	7 (6.9)	0.059	17 (6.7)	8 (9.6)	0.252	0.010	0.337
Treatment with antibiotics and corticosteroids	29 (11.2)	7 (6.9)	0.143	17 (6.7)~~)~~	8 (9.6)	0.252	0.050	0.337

Results expressed as mean ± SD or *n* (%).: BMI, body mass index. P differences between groups analyzed with the χ^2 test (categorical variable); Student's t test (continuous variables) or the Mann–Whitney U test (not-normal distribution in continuous variables). Verified by the Shapiro-Wilk test. p^a: compares BMI < 25 Kg/m^2 vs BMI ≥ 25 Kg/m^2 in each group (CG and IG); p^b: compares women with BMI < 25 Kg/m^2 between groups; p^c, compares women with BMI ≥ 25 Kg/m^2 between groups.
